# Canonical Wnt Pathway Inhibitor ICG-001 Induces Cytotoxicity of Multiple Myeloma Cells in Wnt-Independent Manner

**DOI:** 10.1371/journal.pone.0117693

**Published:** 2015-01-30

**Authors:** Eileen R. Grigson, Maria Ozerova, Alexandra Pisklakova, Hao Liu, Daniel M. Sullivan, Yulia Nefedova

**Affiliations:** 1 H. Lee Moffitt Cancer Center and Research Institute, Tampa, Florida, United States of America; 2 The Wistar Institute, Philadelphia, Pennsylvania, United States of America; Van Andel Institute, UNITED STATES

## Abstract

Canonical Wnt signaling has been implicated in the regulation of multiple myeloma (MM) growth. Here, we investigated whether the targeting of this pathway with a novel pharmacological inhibitor ICG-001 would result in an anti-tumor effect and improvement of chemosensitivity in MM. As expected, ICG-001 specifically down-regulated β-catenin/TCF-mediated transcription in MM cells. Treatment with ICG-001 resulted in growth arrest and apoptosis in MM cell lines and primary MM cells. Moreover, ICG-001 enhanced the cytotoxic effects of doxorubicin and melphalan and abrogated chemoresistance of MM cells to these chemotherapeutics induced by bone marrow stroma. The cytotoxic effect of ICG-001 was caspase-dependent and mediated through transcriptional up-regulation of BH3-only pro-apoptotic members of the Bcl-2 family Noxa and Puma but not through inhibition of canonical Wnt signaling. ICG-001 selectively induced apoptosis in primary MM cells but did not affect non-MM cells of the bone marrow microenvironment. Experiments using a xenograft model of MM showed substantial anti-tumor effects of this compound *in vivo*. Thus, our study demonstrated that the small molecule inhibitor ICG-001 has strong anti-MM effects and could be developed further for therapeutic intervention in this disease.

## Introduction

Multiple myeloma (MM) is a blood cancer characterized by the uncontrolled clonal proliferation of malignant plasma cells that expand and accumulate in the bone marrow (BM). Despite the availability of novel therapeutics including bortezomib and lenalidomide, this disease still remains incurable. A number of signaling pathways, including MAPK, PI3K/Akt, MEK, Jak/STAT, and NFκB, among others, have been implicated in the regulation of survival, proliferation, and growth of MM tumors [1]. Recently, an activation of the canonical Wnt pathway and its involvement in regulation of MM cell growth have been reported [2].

Canonical Wnt signaling is initiated by the binding of soluble Wnt ligands to their receptors Frizzled and the low-density lipoprotein receptor-related protein (LRP) 5 or 6 expressed on the surface of MM cells [3]. A key event in the transduction of the Wnt signal inside the cell is the stabilization of cytoplasmic β-catenin. Under unstimulated conditions, β-catenin is recruited into a destruction complex that also includes axin, adenomatous polyposis coli (APC), and glycogen synthase kinase-3β (GSK3β). This complex facilitates phosphorylation of β-catenin by casein kinase I and GSK3β and targets it for ubiquitination and proteasomal degradation [4]. Binding of Wnts to their receptors results in the inhibition of Axin/APC/GSK3β complex activity and blocking of β-catenin phosphorylation followed by the accumulation of non-phosphorylated β-catenin in the cytoplasm and its consequent translocation to the nucleus. In the nucleus, β-catenin forms a complex with members of the T-cell factor/lymphoid enhancer-binding factor (TCF/LEF) family of transcriptional factors [4] and subsequently recruits transcriptional co-activators including cAMP response element-binding (CREB)-binding protein (CBP) or its homolog p300 as well as other components of the transcription machinery. Together, these events result in turning TCF/LEF from transcriptional repressor into transcriptional activator, thereby initiating transcription of Wnt downstream target genes [5].

MM cell lines and primary MM cells have been shown to overexpress components of canonical Wnt signaling, including the Wnt receptors Frizzled and LRP5/6 and the non-phosphorylated form of β-catenin, suggesting the activation of this pathway. This was in contrast to normal primary plasma cells isolated from human tonsils where the level of β-catenin was undetectable [6, 7]. MM cells were able to respond to stimulation with Wnt3a by up-regulation of the β-catenin level. Both stimulation with Wnt3a and overexpression of the constitutively active form of β-catenin (S33Y) resulted in increased MM cell proliferation [2]. Targeting of β-catenin with a small interfering RNA *in vivo in a mouse model* significantly inhibited the growth of MM tumors [8]. Taken together, these data indicated that blocking of canonical Wnt signaling could represent an attractive approach for MM treatment.

Recently, a novel small molecule selective inhibitor of the Wnt/β-catenin pathway, ICG-001, has been discovered [9]. ICG-001 binds specifically to the transcriptional coactivator CBP, disrupting the interaction of CBP with β-catenin and thus suppressing the Wnt/β-catenin mediated gene transcription. Although the anti-tumor effect of ICG-001 has been demonstrated in several tumor types, including colon adenocarcinoma cells, squamous cell carcinomas in the salivary glands, and acute lymphoblastic leukemia cells [9–11], the effect of this compound on other tumors and the mechanism by which ICG-001 induces apoptosis remain to be clarified. Here, we investigated the effect of ICG-001 in MM. We found that ICG-001 induced apoptosis in MM cells but not in surrounding cells of the BM microenvironment, enhanced the cytotoxic effects of conventional therapeutics in MM, and overcame BM stroma-mediated chemoresistance of MM cells. We also demonstrated that the cytotoxic effect of ICG-001 was independent of inhibition of canonical Wnt signaling and was mediated through transcriptional up-regulation of BH3-only members of the Bcl-2 family (Noxa and Puma). Finally, we found that this small molecule inhibitor of β-catenin/CBP interaction was effective in a mouse xenograft models of MM. Taken together, our data suggested that ICG-001 has therapeutic potential and could be further developed for MM treatment.

## Materials and Methods

### Cell cultures and reagents

Human MM NCI-H929, U266, MM1S, and RPMI-8226 cell lines were obtained from ATCC (Manassas, VA). RPMI-8226-dsRed2 cells expressing dsRed2 fluorescent protein were kindly provided by Dr. Ariosto Silva (H. Lee Moffitt Cancer Center) [12]. Cells were cultured in RPMI-1640 medium supplemented with 10% fetal bovine serum and 1% penicillin-streptomycin-glutamine solution (Invitrogen, Life Technologies, Grand Island, NY). H929 cells were also supplemented with 0.05 mM 2-mercaptoethanol. Doxorubicin and melphalan were purchased from Sigma (St. Louis, MO), pan-caspase inhibitor z-VAD-FMK from Bachem (Torrance, CA), and human recombinant Wnt3a was obtained from R&D Systems (Minneapolis, MN). For *in vitro* studies, ICG-001 (S1 Fig.) was purchased from Selleck Chemicals (Houston, TX) and dissolved in DMSO. For *in vivo* studies, a sodium phosphate form of ICG-001 was synthesized by the Chemical Biology Core Facility of the H. Lee Moffitt Cancer Center.

### Isolation of primary MM cells

This study was approved by the University of South Florida Institutional Review Board protocol. Written informed consent for the use of BM aspirates was obtained from all patients. BM samples were collected from patients with MM. BM mononuclear cells were isolated using Ficoll-Paque gradient centrifugation and incubated with CD138-MicroBeads followed by magnetic separation of positive cells using MidiMACS system according to the manufacturer protocol (Miltenyi Biotec Inc, San Diego, CA). Cells were cultured in -minimal essential medium supplemented with 10% fetal bovine serum and 1% antibiotic/antimycotic. Bone marrow stroma (BMS) was generated from BM mononuclear cells as described earlier [13].

### Flow cytometry

Apoptosis of MM cells was detected by Annexin V binding assay using a LSR II flow cytometer (BD Biosciences, San Jose, CA). Briefly, MM cells were collected, washed twice with ice-cold PBS and once with binding buffer, and stained with FITC- or APC-conjugated Annexin V and DAPI. A minimum of 10,000 events were acquired. Data were analyzed using FlowJo software (Tree Star Inc, Ashland, OR).

For detection of cleaved caspase 3, mononuclear cells isolated from BM of patients with MM were incubated with ICG-001 for 24 hours and then collected and fixed using Cytofix/Cytoperm (BD) according to the manufacturer’s protocol. Cells were then labeled with antibodies against CD138, κ/λ light chain (both from BD), and cleaved caspase 3 (Cell Signaling Technology, Danvers, MA). MM cells were defined as CD138 and κ/λ double positive cells. Mean fluorescence intensity was determined to evaluate the level of cleaved caspase 3 in gated MM cells and surrounding non-MM CD138-negative and κ/λ-negative BM cells.

For detection of cell cycle distribution, cells were collected, fixed with 70% ethanol, and kept overnight at-20°C. Cells were then washed with PBS and stained with 1 mg/mL propidium iodide in the presence of RNase A. At least 10,000 events were acquired using LSR II flow cytometer. Data were analyzed using ModFit software (Verity Software House, Topsham, ME).

### MTT assay

Cells were treated with various concentrations of ICG-001 for 24 hours. 3-[4, 5-Dimethylthiazol-2-yl]-2, 5-diphenyltetrazolium bromide (MTT) dye (Sigma) was added for the last 4 hours of incubation. Insoluble formazan complexes were pelleted and solubilized with DMSO, and absorbance was measured at 540 nm using a Benchmark Plus microplate spectrophotometer (Bio-Rad, Hercules, CA). The IC50 values were calculated using CalcuSyn software (Biosoft, Cambridge, UK). Each experimental condition was done in triplicate and repeated at least once.

### Dual-Luciferase assay

MM.1S cells were transfected with 5 μg of either Wnt/β-catenin reporter plasmid Super8xTOPFlash or control reporter containing mutant TCF/LEF binding sites Super8xFOPFlash (Addgene, Cambridge, MA) and co-transfected with 100 ng of Renilla luciferase-expressing plasmid pRL-TK (Promega, Madison, WI) using nucleofection technique (Lonza-Amaxa, Cologne, Germany). Cells were then cultured for 24 hours with or without 10 μM ICG-001 or 100 ng/mL Wnt3a. After that time, cells were collected and luciferase activity was measured using a Dual-Luciferase Assay System Kit (Promega, Madison, WI). Luminescence was read on a 20/20n Luminometer (Turner Biosystems, Sunnyvale, CA).

### Real-time PCR

MM cell lines were treated overnight with ICG-001 or vehicle control. Real-time PCR was performed using a CFX-96 Real-Time System C1000 Thermal Cycler (Bio-Rad, Hercules, CA). Power SYBR Green Master Mix (Applied Biosystems, Life Technologies, Grand Island, NY) and the following primers were used: cyclin D1 forward 5′-GGCGGAGGAGAACAAACAGA-3′ and reverse 5′-TGGCACAAGAGGCAACGA-3′; c-Myc forward 5′-TCAGAGGTGCCACGTCTCC-3′ and reverse 5′-TCTTGGCAGCAGGATAGTCCTT-3′; and β-actin forward 5′-CCAAGGCCAACCGCGAGAAGA-3′ and reverse 5′-CCGGCCAGCCAGGTCCAGAC-3′. Expression of indicated genes was normalized to the expression of the endogenous control gene β-actin. Expression levels of Noxa and Puma were determined using pre-designed primer and probe set, TaqMan Universal PCR Master Mix (Applied Biosystems), and ABI HT7600 instrument. The qPCR TaqMan Array Human Apoptosis Pathway (Applied Biosystems) was utilized to detect the expression of genes involved in regulation of apoptosis in MM cells treated with ICG-001 or vehicle control.

### Western blotting

Western blotting was performed according to the standard protocol. Membranes were blocked in 5% milk for 1 hour at room temperature and then incubated overnight at 4°C with the primary antibodies against bcl-2, Puma, β-catenin, Myc-Tag, and cleaved caspase 3 (Cell Signaling Technology, Danvers, MA); bcl-xL (BD); mcl-1 and β-actin (Santa Cruz); and Noxa (EMD Millipore, Billerica, MA). Membranes were then washed, incubated with corresponding secondary antibodies, and developed using ECL (Bio-Rad, Hercules, CA).

### Cell transfection

Plasmid encoding human TCF4 pcDNA/Myc-TCF4 was obtained from Addgene (Cambridge, MA). siRNA against β-catenin and control non-targeting pool siRNA were purchased from Dharmacon. U266 and MM1S MM cells were transfected using a nucleofector device (Amaxa-Lonza). MM1S cells overexpressing TCF4 or empty pcDNA3 vector were selected using G418.

### In vivo studies

This study was approved and all experimental procedures were performed in accordance with the guidelines of the University of South Florida and The Wistar Institute Institutional Animal Care and Use Committee. SCID-beige (*C*.*B-Igh-1b/GbmsTac-Prkdc* ^*scid*^-*Lyst* ^*bg*^ *N7*) mice were purchased from Taconic (Germantown, NY) and NSG (*NOD*.*Cg-Prkdc* ^*scid*^ *Il12rg* ^*tm1Wjl*^/*SzJ*) mice were purchased from The Jackson Laboratory (Bar Harbor, ME). Mice were kept in pathogen-free conditions in the animal facility of the Moffitt Cancer Center or The Wistar Institute. 6–8 week old mice were used for experiments. MM tumors were established in SCID-beige mice by subcutaneous inoculation of 1x10^7^ MM RPMI-8226 cells in the right flank. In approximately 3 weeks, when tumors became measurable, mice were assigned to vehicle control or ICG-001-treated group. ICG-001 was administered intraperitoneally twice per day at a dose of 100 mg/kg for 3 weeks. Tumor size was measured twice per week using caliper. Tumor volume was calculated using the following formula: volume = (width)^2^ x length/2. MM tumors were established in NSG mice by intravenous into tail vein inoculation of 5x10^6^ RPMI-8226-dsRed2 cells. Two weeks after tumor cell inoculation mice were randomly assigned to 2 groups and treated either with vehicle control or ICG-001 (twice a day; 100 mg/kg) until termination of the experiment. Tumor burden was evaluated by measuring dsRed2 fluorescence intensity using IVIS 200 In Vivo Imaging System. Mice were euthanized at humane end point.

### Statistical analysis

Statistically significant differences observed in cells treated with single drug compared to cells treated with drug combination were determined using the Student *t* test. P values less than 0.05 were considered significant. Differences in tumor growth *in vivo* were analyzed using two-way ANOVA. GraphPad Prism 5 software was used to perform statistical analysis.

## Results

### Effect of ICG-001 on canonical Wnt signaling

**Fig 1:**
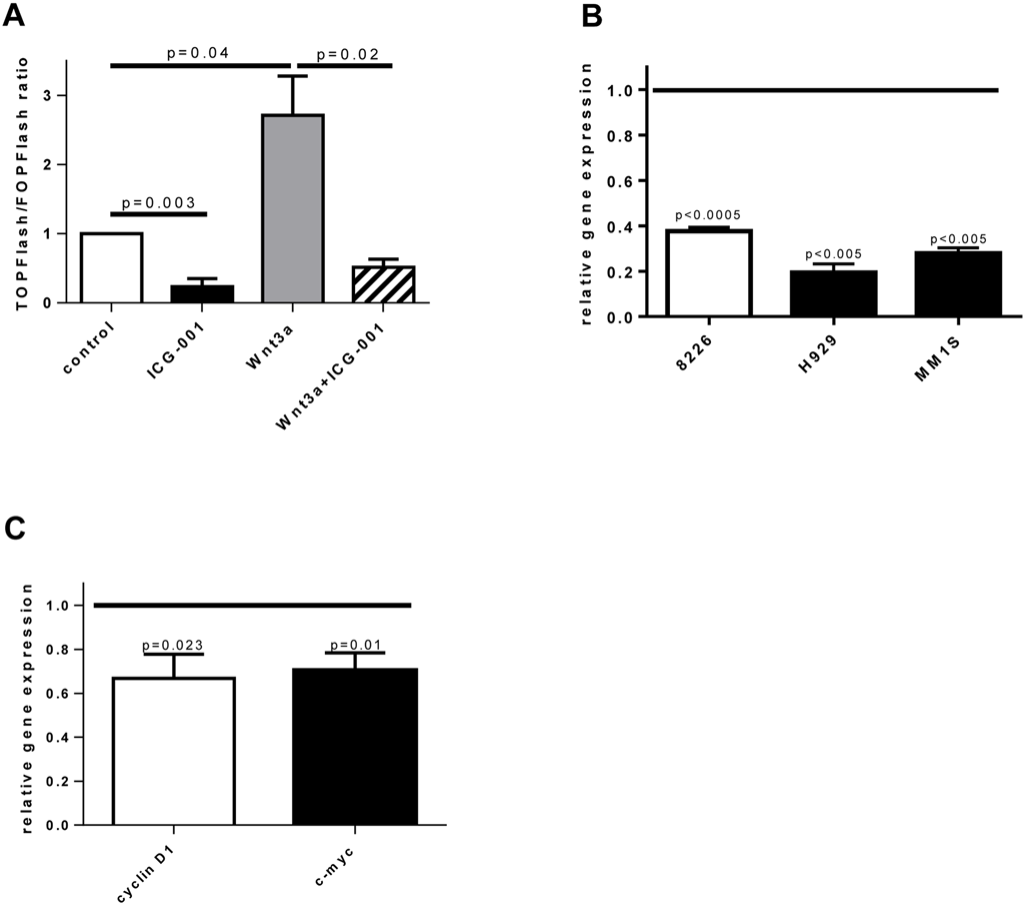
Treatment with ICG-001 inhibits canonical Wnt signaling in MM cells. (**A**) MM1.S cells were transiently transfected with TOPFlash or FOPFlash reporter plasmids and co-transfected with pRL-TK vector. Cells were untreated (control) or treated for 24 hours with ICG-001 (10 μM), Wnt3a (100 ng/mL) or Wnt3a and ICG-001 followed by dual luciferase reporter assay. Combined data from 2 independent experiments are shown. Statistically significant differences are indicated. (**B, C**) MM cell lines (**B**) or primary CD138^+^ MM cells isolated from BM of patients with MM (**C**) were treated with vehicle control or ICG-001 (2.5 μM for RPMI-8226 cells and 10 μM for other cells) for 24 hours. Expression of indicated genes was determined by real-time PCR. Gene expression in vehicle-treated control cells is indicated by the horizontal line. Combined data from 4 independent experiments (**B**) and combined results for primary MM cells obtained from 4 MM patients (**C**) are shown.

Initially, experiments were performed to evaluate the ability of ICG-001 to specifically inhibit canonical Wnt signaling in MM cells. For that, MM MM1.S cells were transfected with TCF reporter or control luciferase plasmids and then treated with ICG-001. As a positive control, MM cells cultured in the presence of Wnt3a, a known activator of canonical Wnt signaling, were used. Addition of Wnt3a induced significant up-regulation of the canonical Wnt pathway while treatment with ICG-001 resulted in inhibition of TCF reporter activity (Fig. 1A). To further confirm the specificity of canonical Wnt signaling inhibition, we evaluated the effect of ICG-001 on the expression of Wnt target gene c-Myc. ICG-001 caused significant down-regulation of c-Myc expression in all MM cell lines studied (Fig. 1B). A similar effect of ICG-001 on expression of Wnt target genes c-myc and cyclin D1 was observed in primary CD138^+^ MM cells isolated from BM of patients with MM (Fig. 1C). Taken together, these data indicated that ICG-001 specifically inhibited canonical Wnt signaling in MM cells.

### Effect of ICG-001 on viability of MM cells

**Fig 2:**
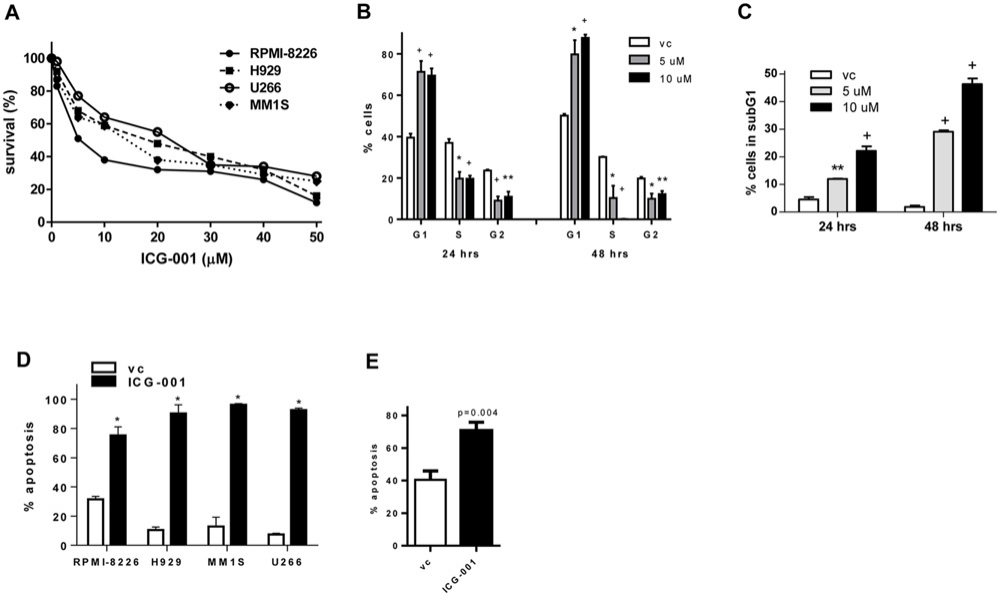
Treatment with ICG-001 decreases viability and induces growth arrest of MM cells. (**A**) MM cells were treated with vehicle control or indicated concentrations of ICG-001 for 24 hours. Cell viability was determined by MTT assay. (**B, C**) MM H929 cells were treated with indicated concentrations of ICG-001 for 24 or 48 hours. Cells were then collected, fixed, and stained with propidium iodide. Cell cycle distribution (**B**) and the proportion of cells in subG1 phase (**C**) were determined. Experiment was performed twice. Results of one experiment performed in triplicates are shown. *P<0.05, **p<0.01, and ^+^p<0.005, as compared to vehicle control group. (**D, E**) Apoptosis of MM cell lines (**D**) or primary CD138^+^ MM cells (**E**) treated with ICG-001 for 24 hours (10 μM for RPMI-8226 and 40 μM for other cell lines) was evaluated using Annexin V binding assay by flow cytometry. In **D**, experiment was repeated 3 times with similar results, with results shown from one representative experiment performed in triplicate. *P<0.000001. In **E**, combined results for primary MM cells obtained from 4 MM patients are shown.

To determine whether ICG-001 has an anti-MM effect, a cellular viability was initially measured using MTT assay in different human MM cell lines. Treatment with ICG-001 resulted in a significant decrease in MM cell viability (Fig. 2A). The following IC_50_ values were established: 6.96±0.14 μM for RPMI-8226 cells, 12.25±2.75 μM for H929 cells, 20.77±0.87 μM for MM.1S cells, and 12.78±0.74 μM for U266 cells. To determine whether inhibition of proliferation and/or induction of cell death were responsible for the observed effect of ICG-001, we analyzed cell cycle distribution and cellular apoptosis. At concentrations sufficient to block Wnt signaling, ICG-001 induced a significant growth arrest, as evident from the accumulation of cells in G0/1 phase and decreased number of cells in S and G2 phases of the cell cycle (Fig. 2B). Interestingly, ICG-001 had also a cytotoxic effect on MM cells, as indicated by the increased proportion of cells accumulated in subG1 phase (Fig. 2C) and confirmed by Annexin V binding assay demonstrating that ICG-001 induced MM cell death through apoptosis (Fig. 2D). A similar cytotoxic effect of ICG-001 was observed in primary CD138^+^ MM cells isolated from BM of patients with MM (Fig. 2E).

### Mechanisms of ICG-001 induced apoptosis in MM cells

**Fig 3:**
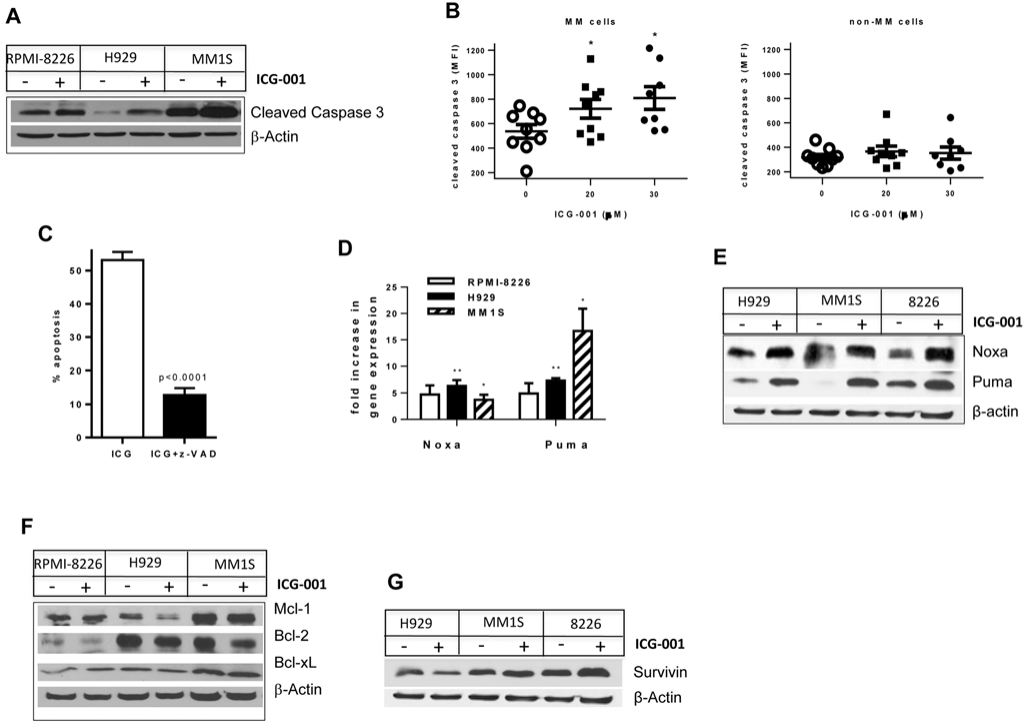
Mechanism of ICG-001-induced apoptosis in MM cells. (**A**) MM cells were treated with ICG-001 (5 μM for RPMI-8226 cells, and 10 μM for H929 and MM1.S cells) 24 hours. Cells were then collected, lysed, and subjected to Western blotting with antibody against cleaved caspase 3. Equal loading was confirmed by re-probing the membrane with β-actin antibody. (**B**) Mononuclear cells were isolated from BM aspirates obtained from patients with MM and were cultured *in vitro* for 24 hours with or without ICG-001. Cells were then collected, labeled with anti-CD138 and anti-λ/κ light chain immunoglobulin antibodies, and stained with antibody against cleaved caspase 3. Level of cleaved caspase 3 was detected by flow cytometry in gated MM and non-MM cell populations. Shown are combined results obtained from BM cells isolated from 9 patients with MM. *P<0.05. (**C**) MM RPMI-8226 cells were treated with 5 μM ICG-001 with or without 100 μM z-VAD for 24 hours. Apoptosis of MM cells was evaluated by Annexin V binding assay using flow cytometry. Experiment was performed twice with similar results. (**D-G**) MM cells were treated with ICG-001 or vehicle control (**D**) overnight or (**E-G**) for 24 hours. (**D**) Expression of indicated genes was determined by real-time PCR and normalized to the expression of β-actin. Shown is fold increase in expression of indicated genes in ICG-001-treated cells over cells treated with vehicle control. Combined results of 3 independent experiments are shown. *P<0.05; **p<0.01. (**E-G**) Western blotting with indicated antibodies was performed. Equal loading was confirmed by re-probing the membranes with antibody against β-actin.

To address the question of how ICG-001 induced apoptosis in MM cells, we first evaluated whether MM cell death was caspase-dependent. MM cell lines were cultured with or without Wnt inhibitor, and the level of cleaved caspase 3 was determined by Western blotting. Treatment with ICG-001 resulted in a significant increase in cleavage of caspase 3 in all MM cell lines tested (Fig. 3A). *Ex vivo* experiments were then performed to confirm the relevance of our *in vitro* data. Mononuclear cells were isolated from BM aspirates obtained from patients with MM and were cultured with or without ICG-001. The level of cleaved caspase 3 was evaluated by flow cytometry in gated populations of BM MM cells and BM non-MM cells. Similar to MM cell lines, inhibition of Wnt signaling with ICG-001 in primary MM cells resulted in significant up-regulation of cleaved caspase 3 (Fig. 3B, left panel), while this compound was not able to induce apoptosis in non-MM cells of BM (Fig. 3B, right panel). Pre-treatment of MM cells with the pan-caspase inhibitor z-VAD abrogated ICG-001 induced apoptosis (Fig. 3C). Taken together, these data indicated that ICG-001 induced apoptosis through a caspase-dependent mechanism.

In order to determine the factor(s) responsible for the anti-MM effect of ICG-001, a human TaqMan Apoptosis Pathway array allowing detection of the expression levels of 92 apoptosis associated genes was performed. Expression of two of them, BH3-only pro-apoptotic members of the Bcl-2 family Noxa and Puma, was found to be up-regulated by ICG-001. Up-regulation of Noxa and Puma was further validated on the gene expression level (Fig. 3D) and confirmed on protein level (Fig. 3E). Treatment of MM cells with ICG-001 did not reduce the protein level of anti-apoptotic Bcl-2 family members Mcl-1, Bcl-2, and Bcl-xL (Fig. 3F). Previous reports have indicated that ICG-001 can induce apoptosis through down-regulation of survivin [9, 11]. Therefore, we investigated whether a decrease in survivin expression could be a mechanism involved in the ICG-001-mediated cytotoxic effect in MM cells. As shown In Fig. 3G, there was no difference in the survivin protein level in MM cells treated or not with ICG-001.

**Fig 4:**
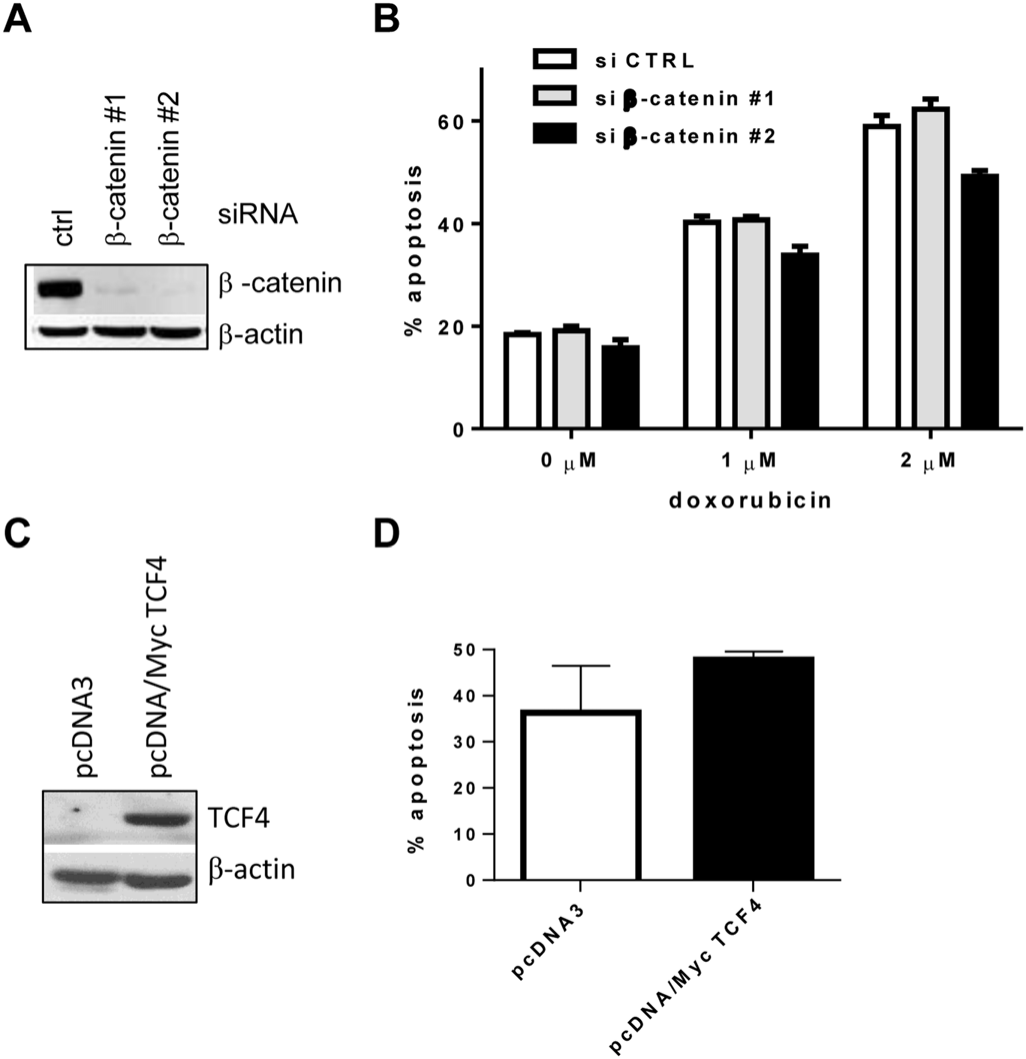
Involvement of Wnt signaling in ICG-001-induced apoptosis in MM cells. **(A, B)** MM MM.1S cells were transfected with siRNA against β-catenin or control non-targeting pool siRNA. (**A**) The efficiency of knockdown was evaluated by Western blotting with antibody against β-catenin. Equal loading was confirmed by re-probing the membrane with antibody against β-actin. (**B**) Cells were then either left untreated (control) or were treated with indicated concentration of doxorubicin for 24 hours. Apoptosis of MM cells was detected by Annexin V/DAPI staining by flow cytometry. Three experiments were performed with similar results. Results of 1 representative experiment are shown. (**C, D**) MM.1S cells were transfected with pcDNA/Myc TCF4 or control empty pcDNA3 plasmid followed by selection of stably transfected clones with G418. (**C**) Overexpression of TCF4 was evaluated by Western blotting using anti-Myc-Tag antibody. Equal loading was confirmed by re-probing the membrane with antibody against β-actin. (**D**) MM.1S cells transfected with pcDNA/Myc TCF4 or empty pcDNA3 vectors were treated with ICG-001 (10 μM) for 24 hours followed by detection of apoptosis by Annexin V binding assay. Combined results of 2 independent experiments are shown.

Previously reported data indicated that ICG-001 induced apoptosis through inhibition of the canonical Wnt pathway [9]. Here, we have addressed the question of whether pro-apoptotic effects of ICG-001 in MM cells are also mediated through the inhibition of canonical Wnt signaling. Beta-catenin is a well-known downstream mediator of this pathway. We used specific siRNA against β-catenin to knock down its expression in MM.1S cells (Fig. 4A). MM cells were then treated with doxorubicin or left untreated. Blocking of Wnt signaling did not result in increased viability of MM cells and did not enhance the effect of doxorubicin in these cells (Fig. 4B). Similar results were obtained in another MM cell line (U266 cells; data not shown).

TCF4 is a transcription factor that mediates the effects of Wnt/β-catenin signaling in MM cells [2]. To further determine a role of inhibition of canonical Wnt signaling in the pro-apoptotic effect of ICG-001 in MM cells, a plasmid encoding human TCF4 was overexpressed in these cells (Fig. 4C) followed by treatment with ICG-001. Forced overexpression of TCF4 was not able to abrogate the pro-apoptotic effect of ICG-001 in MM cells (Fig. 4D), suggesting that the inhibition of canonical Wnt signaling was not responsible for the observed cytotoxicity of ICG-001 in MM cells.

### ICG-001 effect in the context of bone marrow stroma

**Fig 5:**
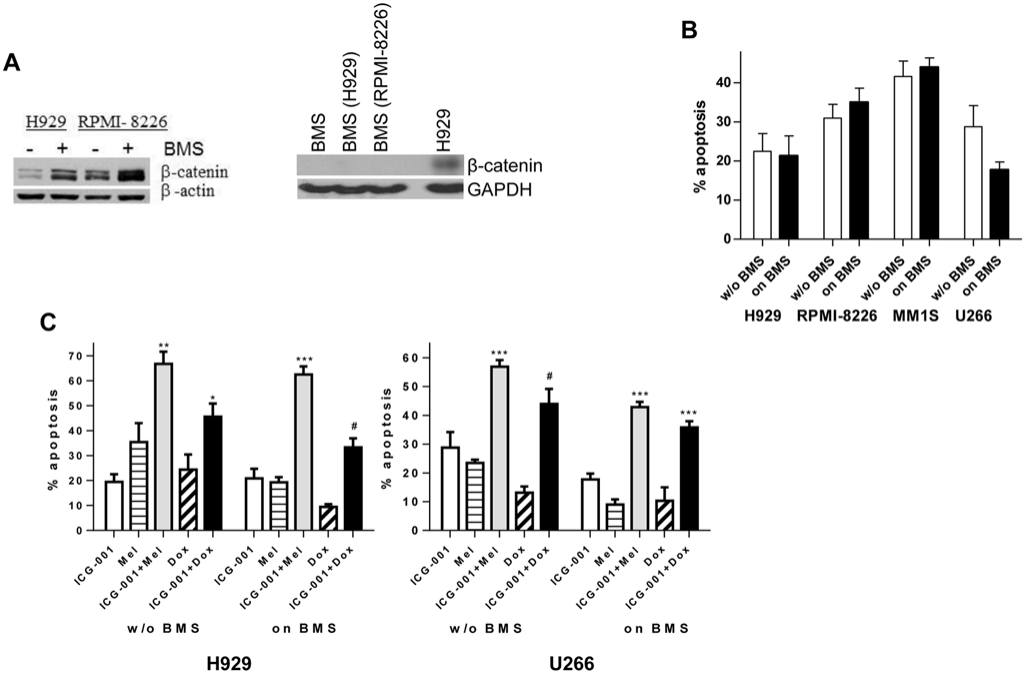
Treatment with ICG-001 overcomes BMS-mediated MM cell chemoresistance. (**A**) MM cells were cultured with or without BMS for 24 hrs. MM cells were then collected and the expression of β-catenin was determined by Western blotting (left panel). BMS cultured alone or with MM cells (indicated in the parenthesis) for 24 hrs were collected and subjected to Western blotting with antibody against β-catenin (right panel). H929 MM cells were used as a positive control for β-catenin expression. (**B**) MM cells cultured on the monolayer of BMS or kept in suspension (without BMS) were treated with ICG-001 or vehicle control for 24 hours. Apoptosis of MM cells was evaluated by Annexin V binding assay. Combined data from 4 independent experiments performed with BMS derived from BM of 4 patients with MM are shown. (**C**) MM H929 or U266 cells were cultured on the top of BMS or kept in suspension (without BMS) with or without 15 μM ICG-001 overnight followed by 24-hour treatment with doxorubicin or melphalan. Apoptosis of MM cells was evaluated using Annexin V binding assay. Shown are values of specific drug-induced apoptosis calculated by subtracting background values (cells treated with vehicle control) from values of drug-induced apoptosis. Combined results from 4 independent experiments performed with BMS derived from BM of 4 patients with MM are shown. *P<0.05, **p<0.005, and ***p<0.001 between ICG-001+chemotherapy combination group and both chemotherapy and ICG-001groups. #p<0.001 between drug combination and doxorubicin group and p<0.05 between drug combination and ICG-001 group.

We have previously reported that BMS protected MM cells from apoptosis induced by chemotherapeutic drugs [14]. We evaluated the activation of Wnt signaling in BMS and MM cells cultured alone or together. Previously published data demonstrated a high variability in β-catenin protein expression level in BMS established from different MM patients or healthy donors [15]. There was no β-catenin expression detected in BMS used for our experiment and cultured with or without MM cells (Fig. 5A, right panel). At the same time, interaction between BMS and MM cells induced activation of canonical Wnt signaling in MM (Fig. 5A, left panel). We investigated whether the presence of BMS altered the sensitivity of MM cells to ICG-001. As shown in Fig. 5B, BMS did not protect all four MM cell lines tested from apoptosis induced by ICG-001. We next evaluated whether ICG-001 would improve the anti-MM effect of conventional chemotherapeutics used for MM therapy. MM cells cultured with or without BMS were treated with ICG-001 overnight followed by the addition of doxorubicin or melphalan for another 24 hours. As expected, BMS protected MM cells from the cytotoxic effects of chemotherapy. Drug-induced apoptosis of MM cells cultured with or without BMS was significantly increased by the addition of ICG-001 (Fig. 5C).

### Effect of ICG-001 in vivo in xenograft model of MM

**Fig 6:**
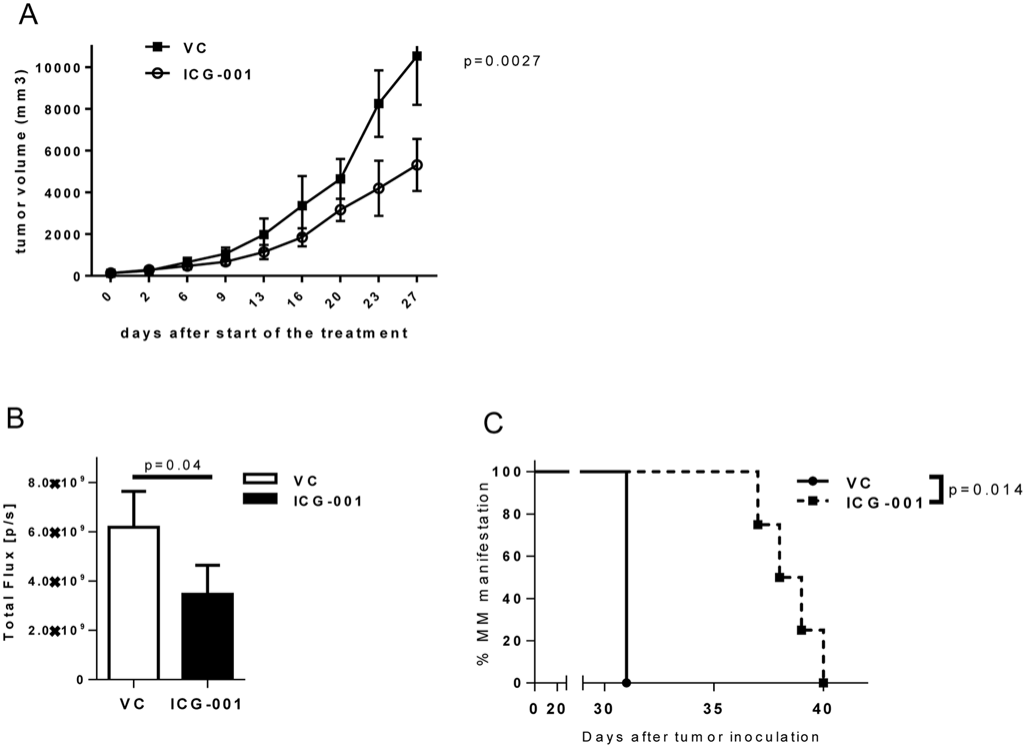
*In vivo* effect of ICG-001 on MM tumor burden. (**A**) MM tumors were established in SCID-beige mice by subcutaneous inoculation of RPMI-8226 cells. In ~ 3 weeks, mice were split into 2 groups (4 mice/group) and treated with either vehicle control (PBS) or 100 mg/kg ICG-001 twice a day. Tumor growth was monitored by measuring tumor size twice a week. Two-way ANOVA was used to analyze the difference between the 2 groups. (**B, C**) MM tumors were established in NSG mice by intravenous inoculation of RPMI-8226-dsRed2 cells. In 2 weeks mice were randomly assigned to 2 groups and treated with either vehicle control (vc; n = 3) or ICG-001 (n = 4). (**B**) Tumor burden was evaluated by measuring dsRed2 protein fluorescence using IVIS 200 In Vivo Imaging System 3 weeks after start of the treatment. (**C**) Onset of hunched posture and hind leg paralysis has been evaluated and compared between groups.

Two xenograft mouse models of MM were used to investigate the anti-tumor effect of ICG-001. In the first model, human RPMI-8226 MM cells were injected subcutaneously into the flank of SCID-beige mice. MM tumor-bearing mice were split into two groups and were given either ICG-001 or vehicle control. As shown in Fig. 6A, the treatment with ICG-001 resulted in significantly reduced MM tumor growth (p = 0.0027). In another model, RPMI-8226-dsRed2 cells were injected intravenously into the tail vein of NSG mice. In this model, MM cells home to the bone marrow that closely resembles human disease. Treatment with ICG-001 resulted in a substantially reduced MM tumor burden as evaluated by the expression of dsRed2 protein (Fig. 6B) and a significantly delayed manifestation of the disease such as hunched posture and hind leg paralysis (Fig. 6C).

## Discussion

Canonical Wnt signaling is one of the key signaling pathways involved in the regulation of cell proliferation, self-renewal, and survival in normal hematopoiesis and different types of cancer [16–19]. Aberrant expression of transcriptionally active β-catenin has been well documented in primary MM cells and MM cell lines [2, 8]. No activating mutations have been identified in MM so far [2], and the mechanism responsible for increased canonical Wnt signaling has yet to be determined. In MM, Wnt/β-catenin signaling has emerged as a critical pathway involved in regulation of the bone disease [20]. Furthermore, its role in MM cell proliferation has been established [2]. However, a contribution of this pathway in the chemosensitivity of tumor cells remains unknown. We and others have previously demonstrated that BMS protect MM cells from chemotherapy-induced apoptosis [14]. We have also found that BMS activate canonical Wnt in these cells (Fig. 5A). Based on these data, a hypothesis was proposed that BMS may mediate MM cell chemoresistance through the activation of Wnt/β-catenin signaling. To address the question of whether canonical Wnt is involved in the regulation of MM cell response to chemotherapeutics, we used ICG-001, a novel small molecule inhibitor of this pathway. As expected, ICG-001 induced growth arrest in MM cells. Treatment with this compound also led to MM cell apoptosis and significantly enhanced the cytotoxic effects of standard chemotherapeutics. The addition of ICG-001 also overcame a chemoprotective effect of BMS on MM cells.

The activation of Wnt/β-catenin has previously been associated with the survival and protection against apoptosis in pre-B mouse lymphocytes and Rat-1 cells [16, 21], and the inhibition of this pathway has been linked with induction of apoptosis mediated through downregulation of survivin or PI3K/AKT pathway [22–25]. Using colon carcinoma cells, Emami et al demonstrated that ICG-001 induced apoptosis through the inhibition of Wnt signaling [9]. Similar data were reported for acute lymphoblastic leukemia cells [11].

To address the question of whether the blocking of canonical Wnt could affect MM cell viability and chemosensitivity, we performed a number of experiments. Knocking down of β-catenin, a downstream mediator of canonical Wnt pathway, neither increased MM cell apoptosis nor enhanced the cytotoxicity of chemotherapeutics in these cells. Overexpression of TCF4, a transcription factor known to mediate Wnt/β-catenin effects in MM cells [2], did not abrogate the cytotoxic effect of ICG-001. Taken together, these results indicated that pro-apoptotic effect of this compound in MM cells was not mediated through the blocking of Wnt/β-catenin signaling. Our data were in agreement with previously published studies demonstrated that activation of Wnt signaling by Wnt3a or overexpression of Wnt inhibitor DKK1 had no effect on MM proliferation and did not sensitize MM cells to apoptosis following treatment with thalidomide or lenalidomide [6, 26, 27]. Blocking of canonical Wnt with a small molecule inhibitor PKF115–585 significantly reduced proliferation and viability of MM cells; however whether this effect was mediated through inhibition of Wnt has not been confirmed [7].

Previous reports indicated that survivin, a protein that inhibits apoptosis, was down-regulated in colon carcinoma and acute lymphoblastic leukemia cells after treatment with ICG-001 [9, 11]. In contrast to these results, ICG-001 did not affect the level of survivin in MM cells. Instead, the expression levels of Noxa and Puma, BH3-only pro-apoptotic members of the Bcl-2 family, were significantly up-regulated after treatment with ICG-001, suggesting that these two proteins were responsible for the pro-apoptotic effect of ICG-001 in MM cells.

Both Puma and Noxa could be transcriptionally regulated through p53-dependent or p53-independent pathways [28]. Although a number of transcriptional factors have been implicated in the p53-independent regulation of these pro-apoptotic Bcl-2 family members, the TCF/LEF family that mediates Wnt effects has previously not been linked to the direct up-regulation of Puma and Noxa. In the same time, Huang et al. demonstrated that knocking down β-catenin resulted in elevated expression of the p53-binding gene TP53BP1 [29]. This gene encodes the 53BP1 protein, which is able to bind wild-type but not mutant p53 protein and enhance p53-mediated transcriptional activity [30]. The pro-apoptotic effects of ICG-001 were similar in MM cells with both wild-type (H929) and mutated p53 (RPMI-8226, U266), suggesting that the 53BP1 protein was not involved in ICG-001 induced apoptosis in MM cells.

CBP functions as a coactivator to a number of transcriptional factors including p53 [31]. Although the specificity of ICG-001 binding to CBP and the disruption of its interaction with β-catenin have previously been demonstrated [9], it does not exclude the possibility of its binding to other transcriptional factors, for example p53, that regulate the expression of Puma and Noxa. These data support our results suggesting the Wnt-independent cytotoxic effect of ICG-001.

We and others have previously shown that the adhesion of tumor cells to BMS results in growth arrest and protection of tumor cells from chemotherapy-induced apoptosis [14, 32]. Although ICG-001 blocked the proliferation of MM cells, the presence of BMS or primary BM cells obtained from patients with MM did not affect its selective cytotoxicity against tumor cells. Moreover, treatment with ICG-001 sensitized MM cells to conventional chemotherapeutics (doxorubicin and melphalan) in the presence and absence of BMS. Taken together, our results indicated that the novel small molecule inhibitor ICG-001 has a strong anti-MM effect and could be considered for further development as a therapeutic agent for the treatment of MM.

## Supporting Information

S1 Fig Chemical structure of ICG-001. (TIF)

## References

[1] Anderson K, Carrasco R (2011) Pathogenesis of myeloma. Annu Rev Pathol 6: 249–274. 10.1146/annurev-pathol-011110-130249 21261519

[2] Derksen P, Tjin E, Meijer H, Klok M, MacGillavry H, et al (2004) Illegitimate WNT signaling promotes proliferation of multiple myeloma cells. Proc Natl Acad Sci U S A 101: 6122–6127. 15067127 10.1073/pnas.0305855101 PMC395933

[3] Qiang Y, Walsh K, Yao L, Kedei N, Blumberg P, et al (2005) Wnts induce migration and invasion of myeloma plasma cells. Blood 106: 1786–1793. 15886323 10.1182/blood-2005-01-0049 PMC1895227

[4] Clevers H, Nusse R (2012) Wnt/b-Catenin Signaling and Disease. Cell 149: 1192–1205. 10.1016/j.cell.2012.05.012 22682243

[5] Takahashi-Yanaga F, Kahn M (2010) Targeting Wnt signaling: can we safely eradicate cancer stem cells? Clin Cancer Res 16: 3153–3162. 10.1158/1078-0432.CCR-09-2943 20530697

[6] Qiang Y, Endo Y, Rubin J, Rudikoff S (2003) Wnt signaling in B-cell neoplasia. Oncogene 22: 1536–1545. 12629517 10.1038/sj.onc.1206239

[7] Sukhdeo K, Mani M, Zhang Y, Dutta J, Yasui H, et al (2007) Targeting the beta-catenin/TCF transcriptional complex in the treatment of multiple myeloma. Proc Natl Acad Sci U S A 104: 7516–7521. 17452641 10.1073/pnas.0610299104 PMC1863489

[8] Ashihara E, Kawata E, Nakagawa Y, Shimazaski C, Kuroda J, et al (2009) β-Catenin Small Interfering RNA Successfully Suppressed Progression of Multiple Myeloma in a Mouse Model. Clin Cancer Res 15: 2731–2738. 10.1158/1078-0432.CCR-08-1350 19351774

[9] Emami K, Nguyen C, Ma H, Kim D, Jeong K, et al (2004) A small molecule inhibitor of β-catenin/cyclic AMP response element-binding protein transcription. Proc Natl Acad Sci U S A 101: 12682–12687. 15314234 10.1073/pnas.0404875101 PMC515116

[10] Wend P, Fang L, Zhu Q, Schipper J, Loddenkemper C, et al (2013) Wnt/β-catenin signalling induces MLL to create epigenetic changes in salivary gland tumours. EMBO J 32: 1977–1989 10.1038/emboj.2013.127 23736260 PMC3715856

[11] Gang E, Hsieh Y, Pham J, Zhao Y, Nguyen C, et al. (2013) Small-molecule inhibition of CBP/catenin interactions eliminates drug-resistant clones in acute lymphoblastic leukemia. Oncogene. 10.1038/onc.2013.169 PMC3994178 23728349

[12] Khin Z, Ribeiro M, Jacobson T, Hazlehurst L, Perez L, et al (2014) A preclinical assay for chemosensitivity in multiple myeloma. Cancer Res 74: 56–67. 10.1158/0008-5472.CAN-13-2397 24310398 PMC3915502

[13] Nefedova Y, Cheng P, Alsina M, Dalton W, Gabrilovich D (2004) Involvement of Notch-1 signaling in bone marrow stroma-mediated de novo drug resistance of myeloma and other malignant lymphoid cell lines. Blood 103: 3503–3510. 14670925 10.1182/blood-2003-07-2340

[14] Nefedova Y, Landowski T, Dalton W (2003) Bone marrow stromal-derived soluble factors and direct cell contact contribute to de novo drug resistance of myeloma cells by distinct mechanisms. Leukemia 17: 1175–1182. 12764386 10.1038/sj.leu.2402924

[15] Qiang Y, Hu B, Chen Y, Zhong Y, Shi B, et al (2009) Bortezomib induces osteoblast differentiation via Wnt-independent activation of beta-catenin/TCF signaling. Blood 113: 4319–4330 10.1182/blood-2008-08-174300 19196662 PMC2676089

[16] Reya T, O’Riordan M, Okamura R, Devaney E, Willert K, et al (2000) Wnt signaling regulates B lymphocyte proliferation through a LEF-1 dependent mechanism. Immunity 13: 15–24. 10933391 10.1016/s1074-7613(00)00004-2

[17] Zhao C, Blum J, Chen A, Kwon H, Jung S, et al (2007) Loss of beta-catenin impairs the renewal of normal and CML stem cells in vivo. Cancer Cell 12: 528–541. 18068630 10.1016/j.ccr.2007.11.003 PMC2262869

[18] Lento W, Congdon K, Voermans C, Kritzik M, Reya T (2013) Wnt Signaling in Normal and Malignant Hematopoiesis. Cold Spring Harb Perspect Biol 5(2). pii: a008011 10.1101/cshperspect.a008011 23378582 PMC3552513

[19] Anastas J, Moon R (2013) WNT signalling pathways as therapeutic targets in cancer. Nat Rev Cancer 13: 11–26. 10.1038/nrc3419 23258168

[20] Edwards C, Edwards J, Lwin S, Esparza J, Oyajobi B, et al (2007) Increasing Wnt signaling in the bone marrow microenvironment inhibits the development of myeloma bone disease and reduces tumor burden in bone in vivo. Blood 111: 2833–2842. 18094333 10.1182/blood-2007-03-077685 PMC2254537

[21] Chen S, Guttridge D, You Z, Zhang Z, Fribley A, et al (2001) WNT-1 Signaling Inhibits Apoptosis by Activating β-Catenin/T Cell Factor—Mediated Transcription. J Cell Biol 152: 87–96. 11149923 10.1083/jcb.152.1.87 PMC2193656

[22] Sun P, Xiong H, Kim T, Ren B, Zhang Z (2006) Positive inter-regulation between beta-catenin/T cell factor-4 signaling and endothelin-1 signaling potentiates proliferation and survival of prostate cancer cells. Mol Pharmacol 69: 520–531. 16291872 10.1124/mol.105.019620

[23] Mazieres J, You L, He B, Xu Z, Lee A, et al (2005) Inhibition of Wnt16 in human acute lymphoblastoid leukemia cells containing the t(1;19) translocation induces apoptosis. Oncogene 24: 5396–5400. 16007226 10.1038/sj.onc.1208568

[24] You L, He B, Xu Z, Uematsu K, Mazieres J, et al (2004) An anti-Wnt-2 monoclonal antibody induces apoptosis in malignant melanoma cells and inhibits tumor growth. Cancer Res 64: 5385–5389. 15289346 10.1158/0008-5472.CAN-04-1227

[25] Veeramachaneni N, Kubokura H, Lin L, Pippin J, Patterson G, et al (2004) Down-regulation of beta catenin inhibits the growth of esophageal carcinoma cells. J Thorac Cardiovasc Surg 127: 92–98. 14752418 10.1016/j.jtcvs.2003.06.008

[26] Colla S, Zhan F, Xiong W, Wu X, Xu H, et al (2007) The oxidative stress response regulates DKK1 expression through the JNK signaling cascade in multiple myeloma plasma cells. Blood 109: 4470–4477. 17255354 10.1182/blood-2006-11-056747 PMC1885505

[27] Qiang Y, Shaughnessy JJ, Yaccoby S (2008) Wnt3a signaling within bone inhibits multiple myeloma bone disease and tumor growth. Blood 112: 374–382. 10.1182/blood-2007-10-120253 18344425 PMC2442747

[28] Shibue T, Taniguchi T (2006) BH3-only proteins: Integrated control point of apoptosis. Int J Cancer. 10.1002/ijc.21751 16572413

[29] Huang M, Wang Y, Sun D, Zhu H, Yin Y, et al (2006) Identification of genes regulated by Wnt/β-catenin pathway and involved in apoptosis via microarray analysis. BMC Cancer 6: 221 16959035 10.1186/1471-2407-6-221 PMC1574340

[30] Iwabuchi K, Bartel P, Li B, Marraccino R, Fields S (1994) Two cellular proteins that bind to wild-type but not mutant p53. Proc Natl Acad Sci U S A 91: 6098–7102. 8016121 10.1073/pnas.91.13.6098 PMC44145

[31] Grossman S (2001) p300/CBP/p53 interaction and regulation of the p53 response. Eur J Biochem 268: 2773–2778. 11358491 10.1046/j.1432-1327.2001.02226.x

[32] Meads M, Gatenby R, Dalton W (2009) Environment-mediated drug resistance: a major contributor to minimal residual disease. Nat Rev Cancer 9: 665–674. 10.1038/nrc2714 19693095

