# Supplementary figures and images for: Canonical Wnt Pathway Inhibitor ICG-001 Induces Cytotoxicity of Multiple Myeloma Cells in Wnt-Independent Manner
Source: PLoS One. 2015 Jan 30;10(1):e0117693. doi: 10.1371/journal.pone.0117693 (PMC4311909; doi:10.1371/journal.pone.0117693)

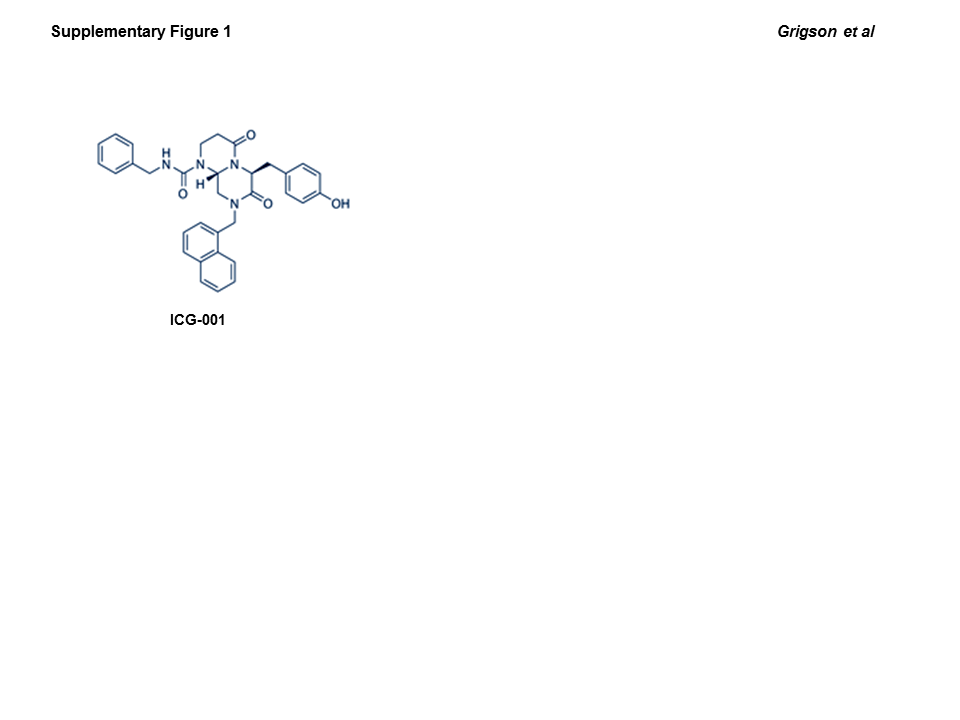

Supplement: S1 Fig — (TIF) [file pone.0117693.s001.tif]
